# Cryptic species in time and space: an assessment of cryptic diversity within eight nominal species of Hydrozoa (Cnidaria)

**DOI:** 10.1098/rspb.2023.0851

**Published:** 2023-08-09

**Authors:** Maria Pia Miglietta, Sarah Pruski

**Affiliations:** Marine Biology, Texas A&M University at Galveston, Galveston, TX 77553-1675, USA

**Keywords:** Hydrozoa, Gulf of Mexico, DNA barcoding, sympatric

## Abstract

Sampling in multiple localities, coupled with molecular barcoding, has shown that nominal species with wide geographical distribution often harbour local cryptic species in allopatry. Cryptic species in sympatry, however, can be easily missed if they have different seasonality, because they can be identified only through long-term frequent sampling (i.e. sampling through time of the same species in the same location). This is especially true in planktonic invertebrates that exhibit strong seasonality. By integrating mitochondrial 16S sequences of eight species of Hydrozoa (Cnidaria) collected weekly for a year in one Gulf of Mexico region, with sequences gathered globally, we investigate the presence of cryptic species within a temporal gradient (regionally) and on a spatial (worldwide) scale. We find that eight species of Hydrozoa are composed of 28 cryptic species, with 16 of them appearing in sympatry but with non-overlapping seasonality. The high number of sympatric cryptic species could only be discovered through extensive and prolonged regional sampling efforts. The bi-dimensional cryptic diversity (in time and space) highlighted in this study is essential for understanding processes of evolution, biogeography dispersal in the sea, and for more realistic biodiversity assessments.

## 1. Introduction

Collecting and identifying organisms remains one of the most expensive enterprises in biology, and high-frequency, long-term local observations are rare. This is true in the marine environment, especially for planktonic marine invertebrates. Planktonic communities are highly variable on a temporal scale [1]. Frequent and long-term sampling is crucial to understanding their ecological and temporal dynamics and essential for estimating biodiversity patterns. The lack of continuous local sampling may hamper our ability to detect cryptic species that exist in sympatry but appear on a temporal gradient.

The discovery of cryptic species in the sea (defined as two or more species classically assigned to a single nominal species because morphologically indistinguishable [2]) has received ample attention in the last 50 years [3–5]. Cosmopolitan nominal species have often been shown to be complexes of local cryptic species with allopatric ranges. However, planktonic species that are morphologically indistinguishable and inhabit the same locality, but show different seasonality have received little to no attention due to the dense long-term sampling that is needed to discover them [1].

In most groups of marine animals, cryptic species can be detected using barcoding techniques [5–8], which are a fast and relatively inexpensive option. Species identification in Hydrozoa (Cnidaria) often relies on a fragment of the mitochondrial 16S rRNA sequences [9–12]. Hydrozoa have a complex life cycle with benthic polyps and planktonic medusae. The production of medusae by the polyps occurs through budding, which happens on a strong seasonal basis and the triggers of which are unknown for most species. Medusae are thus present in the plankton at a specific time of the year only [13]. In this paper, we analyse 467 16S rRNA sequences from eight species of Hydrozoa (phylum Cnidaria) commonly found in the Gulf of Mexico (GoM) and worldwide, all with motile planktonic stages. Using a barcoding approach, we investigate the presence of cryptic species through time (in Galveston Bay, GoM) and space (worldwide).

## 2. Methods

We analyse the mitochondrial 16S phylogeny of 8 species commonly found in the GoM, namely *Liriope tetraphylla, Lovenella assimilis, Nemopsis bachei, Eucheilota maculata, Ectopleura dumortierii, Malagazzia carolinae, Blackfordia virginica* and *Obelia dichotoma*. Sequences belonging to these eight species were obtained from medusae collected weekly along a 12-month series in the GoM (published in [14] and available on GenBank). Medusae were collected using a 100-micron net, 90 cm long, and a 30 cm mouth, on Pelican Island (29°18′47.0″ N, 94°48′59.8″ W), within the boat basin at Texas A&M University at Galveston. Two tows per day were conducted three to four times a week, from September 2015 to September 2016 (see [14]). Medusae were identified to the species level using various taxonomic keys (e.g. [15]). Once sequences were produced, identification was confirmed by comparing them against the BLAST dataset on GenBank.

For each species, sequences from Pruski & Miglietta [14] were assembled with all conspecific 16S gene sequences available on GenBank (see electronic supplementary material, table S1, for a list of sequences and their accession number). The eight datasets (one for each species) were assembled and aligned using Geneious 10.1.3. Alignments were checked by eye, and phylogenetic trees were built using maximum-likelihood and Bayesian inference in RaxML and MrBayes as implemented in Topali v2.5. The best-fit model was automatically calculated by Topali v2.5 and implemented in the phylogenetic analyses. A list of specimens for each dataset, including their GenBank accession number and the locality where they were collected, can be found in the electronic supplementary material, table S1. ML bootstraps were calculated in RaxML on 500 bootstrap runs. Bayesian posterior probability was calculated using the following parameters: number of runs, 2; number of generations, 100 000; sample frequency, 10; burn-in, 25%. All trees are rooted at midpoint. Distance between clades (p-distance) and intraclade genetic divergence were calculated in Mega11 [16]. Phylogenetic trees were visualized in Figtree v1.4.4.

**Figure 1.**
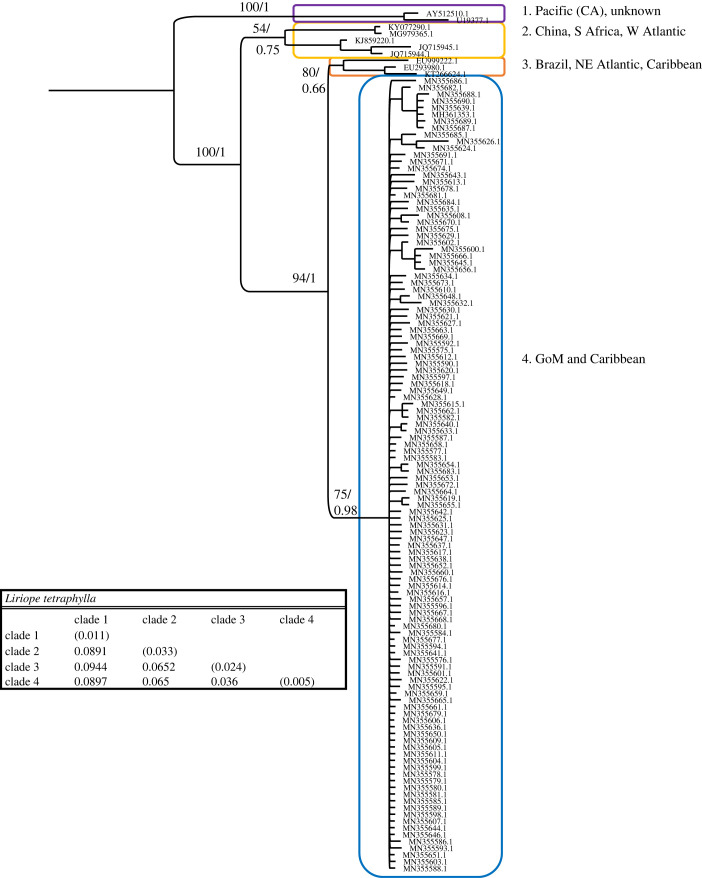
Phylogenetic hypothesis based on the mitochondrial 16S gene of *Liriope tetraphylla.* Numbers at nodes indicate bootstrap values and posterior probabilities (not reported when less than 60%). Genetic distance between clades reported in table at the bottom.

**Figure 2.**
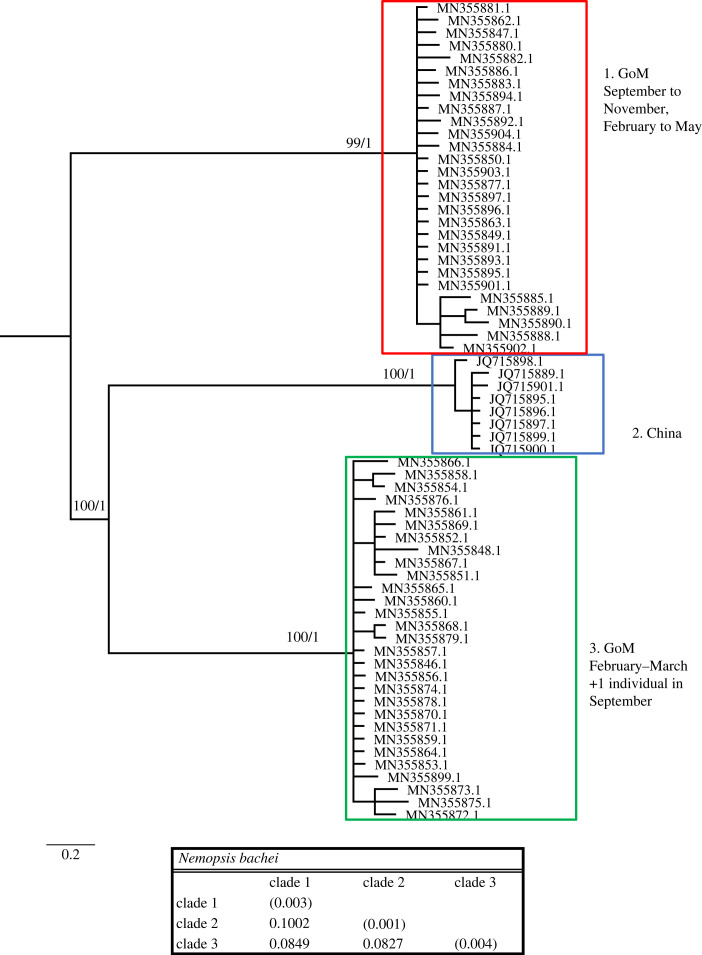
Phylogenetic hypothesis based on the mitochondrial 16S gene of *Nemopsis bachei*. Numbers at nodes indicate bootstrap values and posterior probabilities (not reported when less than 60%). Genetic distance between clades reported in table at the bottom.

**Figure 3.**
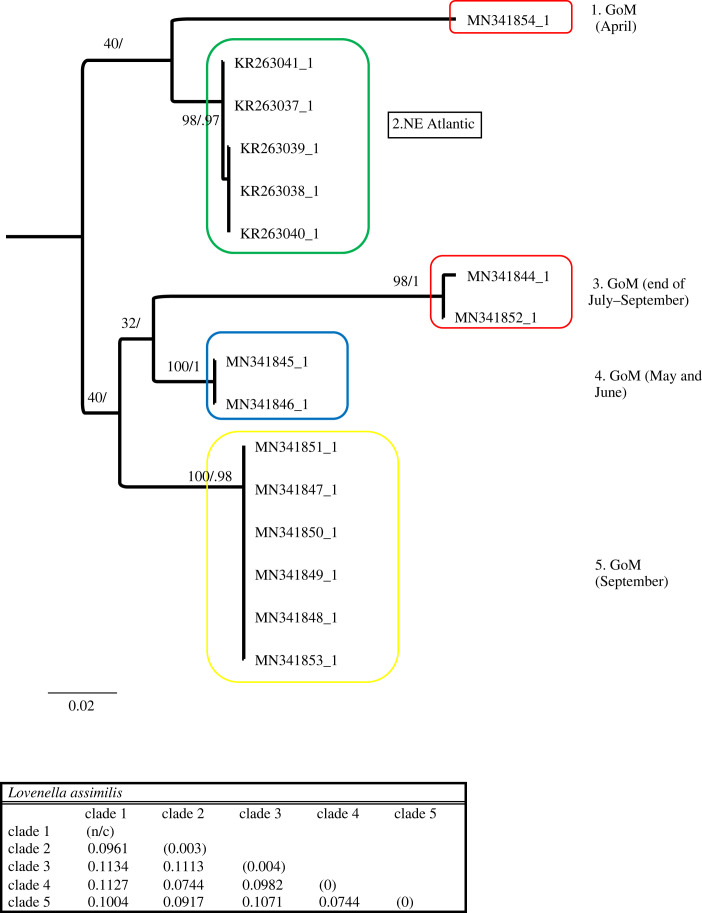
Phylogenetic hypothesis based on the mitochondrial 16S gene of *Lovenella assimilis*. Numbers at nodes indicate bootstrap values and posterior probabilities (not reported when less than 60%). Genetic distance between clades reported in table at the bottom.

**Figure 4.**
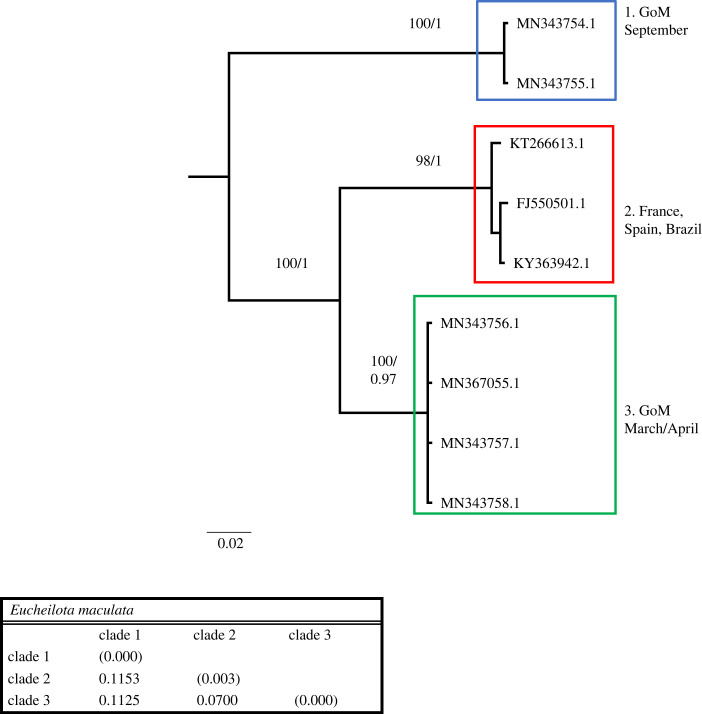
Phylogenetic hypothesis based on the mitochondrial 16S gene of *Eucheilota maculata*. Numbers at nodes indicate bootstrap values and posterior probabilities (not reported when less than 60%). Genetic distance between clades reported in table at the bottom.

**Figure 5.**
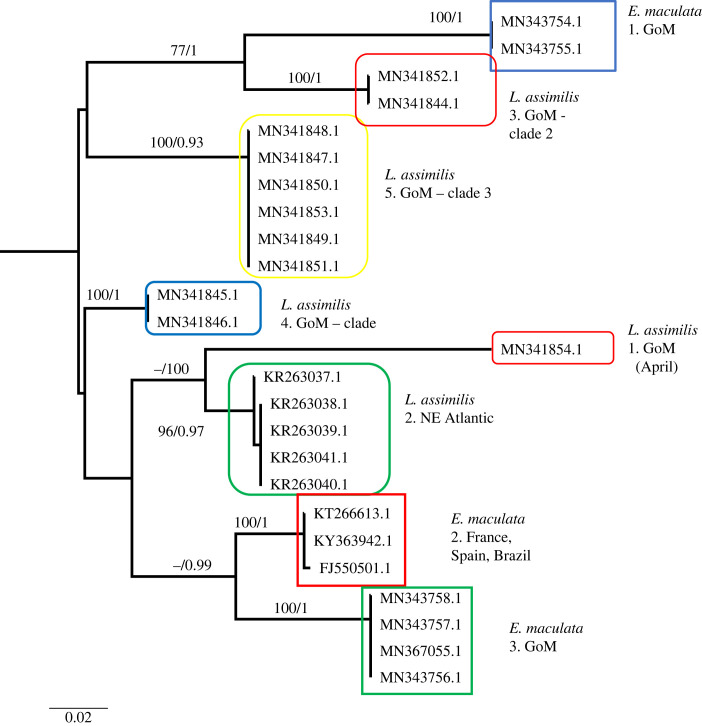
Phylogenetic hypothesis based on the mitochondrial 16S gene of *Lovenella assimilis* and *Eucheilota maculata* (combined). Numbers at nodes indicate bootstrap values and posterior probabilities (not reported when less than 60%). Genetic distance between clades reported in table at the bottom.

**Figure 6.**
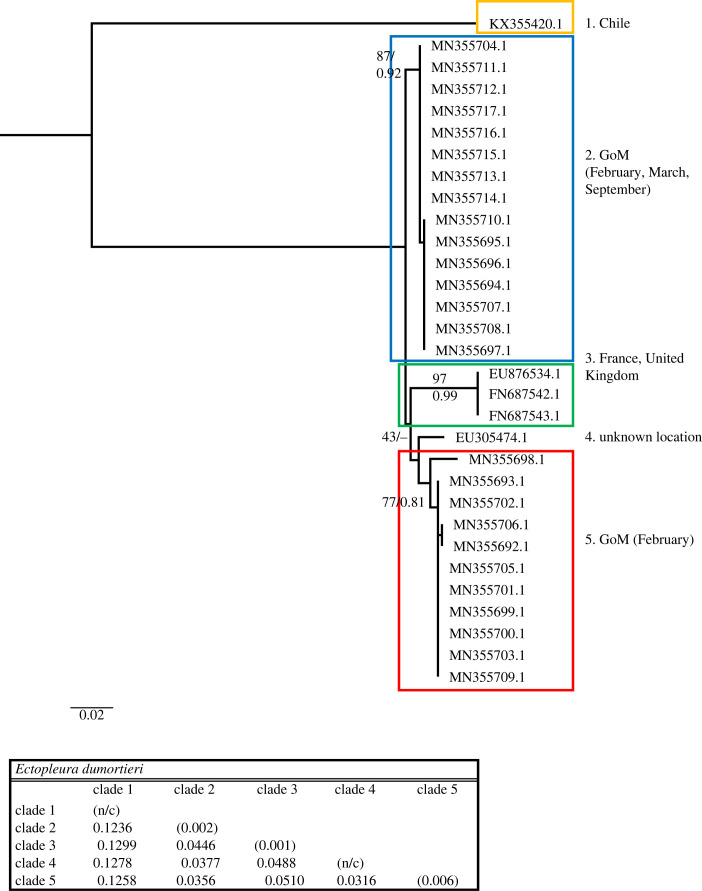
Phylogenetic hypothesis based on the mitochondrial 16S gene of *Ectopleura dumortierii*. Numbers at nodes indicate bootstrap values and posterior probabilities (not reported when less than 60%). Genetic distance between clades reported in table at the bottom.

**Figure 7.**
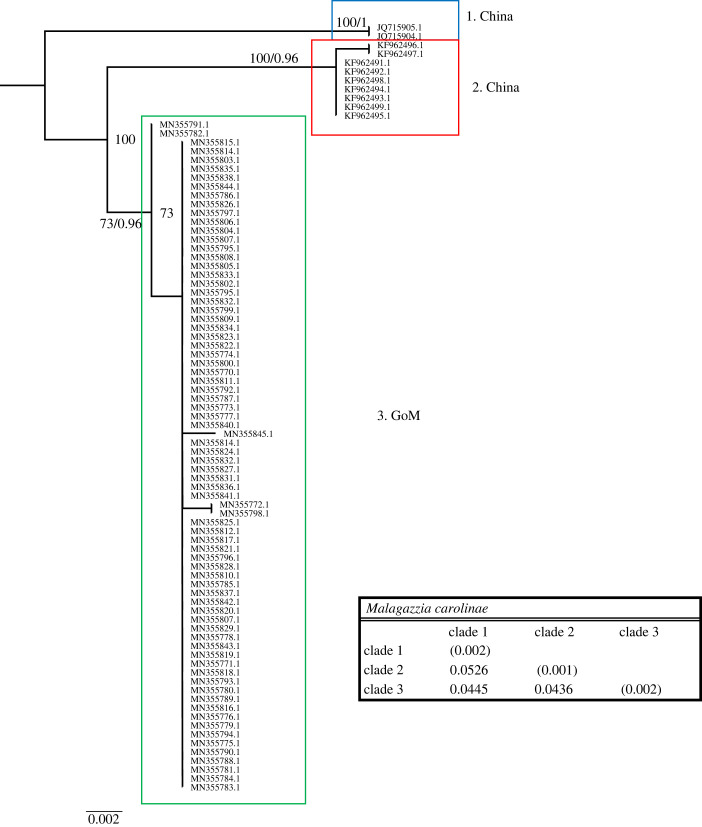
Phylogenetic hypothesis based on the mitochondrial 16S gene of *Malagazzia carolinae.* Numbers at nodes indicate bootstrap values and posterior probabilities (not reported when less than 60%). Genetic distance between clades reported in table at the bottom.

**Figure 8.**
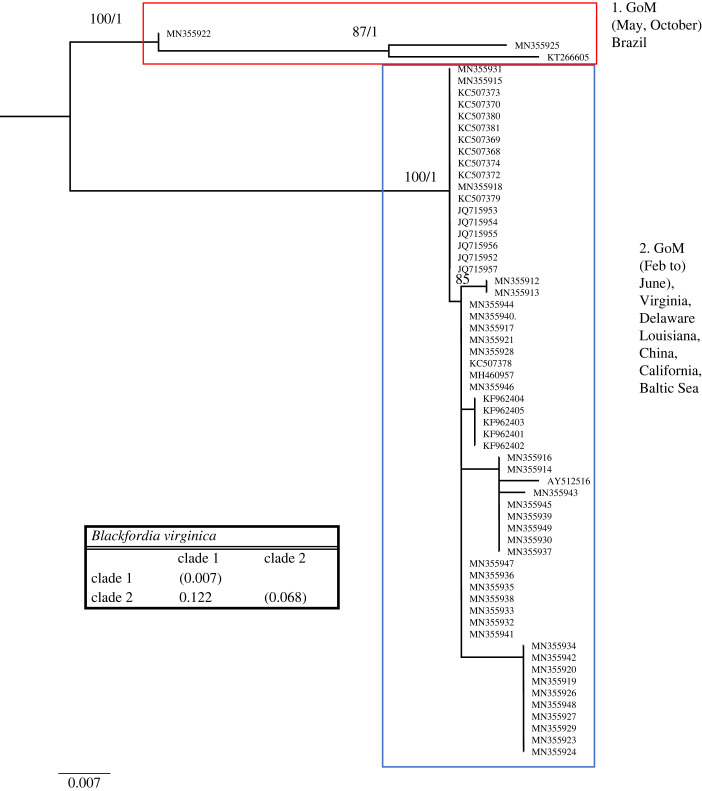
Phylogenetic hypothesis based on the mitochondrial 16S gene of *Blackfordia virginica*. Numbers at nodes indicate bootstrap values and posterior probabilities (not reported when less than 60%). Genetic distance between clades reported in table at the bottom.

**Figure 9.**
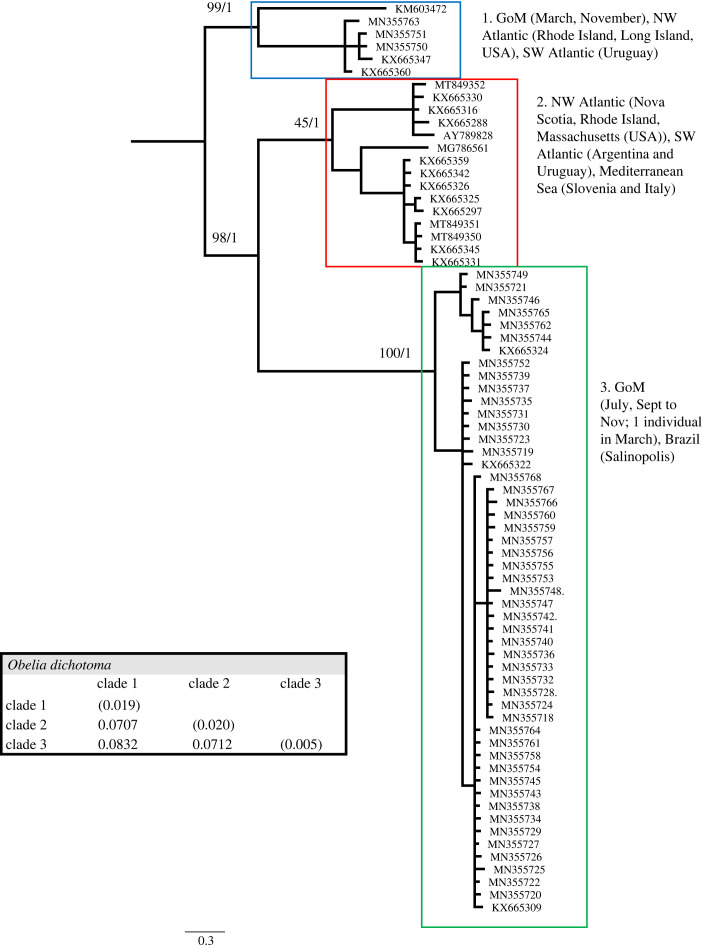
Phylogenetic hypothesis based on the mitochondrial 16S gene of *Obelia dichotoma*. Numbers at nodes indicate bootstrap values and posterior probabilities (not reported when less than 60%). Genetic distance between clades reported in table at the bottom.

Clades shown in figures 1–9 were visually identified on the phylogenetic trees by considering bootstrap/posterior probabilities values and distance between clades (with a threshold set at 3.0% [17–20]). Species delimitation analyses were computed using three different methods. First, we performed automatic barcode gap discovery (ABGD) analyses [21] implemented through the Web server (bioinfo.mnhn.fr/abi/public/abgd/). ABGD analyses were based on the K2P model, with a minimum intraspecific variability Pmin = 0.001, maximum intraspecific variability Pmax = 0.1 and minimum gap width× = 1.5. A second set of species delimitation analysis was performed using the assemble species by automatic partitioning (ASAP) method [22] implemented through the Web server (https://bioinfo.mnhn.fr/abi/public/asap/). The K80 (Kimura) model was used to compute distances. All other parameters were left at default values. With the ASAP delimitation, we considered both the partitions with first and second-best ASAP score, following recommendations of Puillandre *et al*. [22]. Finally, for tree-based methods, we used the Species Delimitation plugin for the Geneious bioinformatic software [23]. The Species Delimitation plugin in Geneious tests different species delimitations that are defined *a priori* [23]. We used the clades inferred in our phylogenetic analysis (shown in figures 1–9), recalculated in Geneious built-in tree builder, as a guide to calculate the following parameters: P ID(strict) and P ID(liberal) defined as the mean (95% confidence interval) probability of correctly identifying an unknown member of the putative species using the criterion that it falls within (strict), or sister to (liberal), the species clade in a tree [23]; Rosenberg's P_AB_, a test for taxonomic distinctiveness based on the null hypothesis that monophyly is a chance outcome of random branching; and Rodrigo's P(Randomly Distinct), which is the probability that a clade has the observed distinctiveness due to random coalescence (P(RD) of 0.005 or less suggests cryptic species) [24].

## 3. Results

The *Liriope tetraphylla* dataset consisted of 128 sequences and 651 nucleotides, of which 328 (50.38%) were phylogenetically informative. *L. tetraphylla* was present in all four seasons in the GoM, February through May, July and September. GoM sequences form a single well-supported and homogeneous clade, also comprising one sequence from Bocas del Toro, Panama (Caribbean Sea) (figure 1, clade 4). Sister to the GoM clade is a small clade composed of three sequences from Brazil, the Caribbean Sea and the North Atlantic/Mediterranean region (precise localities were not reported in GenBank). An additional clade with low support comprises sequences from China, South Africa and the West Antarctica Peninsula. Finally, a fourth clade contains sequences from California and an unknown locality*.* Overall, *L. tetraphylla* is composed of four clades. Distances between the four clades range from 3.6% to 9.4% (figure 1).

The *Nemopsis bachei* dataset included 65 sequences (8 from China and 57 from the GoM) and 650 nucleotides, of which 264 (41.23%) were phylogenetically informative. *Nemopsis bachei* sequences from the GoM clustered in two distinct and well-supported clades (figure 2), one found in September to November and February to May, and one found predominantly in February and March, with one single individual found in September (clades 1 and 3 in figure 2). A third well-supported clade includes eight sequences exclusively from China. Distances between the three clades range from 8.29% to 10.02%.

The *Lovenella assimilis* dataset consisted of 16 sequences, of which 11 were from the GoM and five from the northeast Atlantic, and 622 nucleotides, of which 192 (30.87%) were phylogenetically informative. *Lovenella assimilis* phylogenetic tree (figure 3) shows three distinct lineages in the GoM, one found in May and June (clade 4), one in July and September (clade 3) and one in September only (clade 5). One single specimen found in the GoM in April diverged significantly, showing a possible fourth lineage (clade 1). To these primarily GoM clades, we add another well-supported clade from France and the English Channel (clade 2). Overall, *L. assimilis* is composed of five clades. Distances between these clades range from 7.44% to 11.34%.

The *Eucheilota maculata* dataset included nine sequences, of which six were from the GoM, and 621 nucleotides, of which 129 (20.77%) were phylogenetically informative. The phylogenetic tree shows *E. maculata* (figure 4) is composed of three monophyletic clades. The GoM specimens form two well-supported clades, one found only in September and one in March and April. Within these clades is a third from France (Normandy), Spain (Galicia) and Brazil (San Sebastiao). Genetic distances between these three clades range from 7.00% to 11.53%.

Because *Eucheilota* and *Lovenella* have a history of confusing taxonomy with some evidence that species assigned to the two genera may belong to the same species (see [25]), the two datasets were merged and analysed as one. The resulting dataset comprised 25 sequences (9 sequences identified as *E. maculata* and 16 as *L. assimilis*), 573 sites, of which 171 (29.84%) were phylogenetically informative. The tree of the combined datasets is shown in figure 5. We can identify an *E. maculata* clade containing specimens from the GoM and France, Spain and Brazil. However, two sequences from GoM (clade 1 in figure 4) are nested within *L. assimilis*. While all clades represented in figure 5 have good bootstrap support and posterior probabilities, the relationship between clades is unresolved.

The *Ectopleura dumortieri* dataset included 31 sequences, of which 26 were from the GoM, and 569 nucleotides, of which 170 (29.88%) were phylogenetically informative. *E. dumortieri* phylogenetic trees showed multiple lineages (figure 6), with two found primarily in the GoM: one clade contains all GoM sequences from specimens collected in February, March and September, and the second collected in February. A third clade comprises sequences from France and the United Kingdom (English Channel, eastern Atlantic Ocean). This third clade and the February GoM clade form a monophyletic group with an additional sequence (GenBank EU305474) from an unknown locality. Additionally, a single sequence from Chile (eastern Pacific Ocean) shows a remarkable genetic distance (ranging around 12%) from all the other *E. dumortieri* lineages. In total, *E. dumortieri* shows five clades with distances ranging from 3.1% to 12.99%.

The *Malagazzia carolinae* dataset included 87 sequences, 76 from the GoM and 11 from China, 638 nucleotides, 241 (37.93%) being phylogenetically informative. *M. carolinae* was collected in the GoM in spring, autumn and summer; all sequences fall within one clade (figure 7). Sequences from China form two additional, well-supported clades. The genetic distance between the three clades ranges between 4.3 and 5.2%.

The *Blackfordia virginica* dataset comprised 62 sequences, of which 38 were from the GoM, and 648 nucleotides, 256 (39.51%) being phylogenetically informative. *Blackfordia virginica* phylogenetic tree shows two well-supported clades (figure 8). One clade is composed of individuals from the GoM (Louisiana and Texas) (collected in February through June), northeast Atlantic (Delaware, Virginia, California), Baltic Sea and China. A second clade comprises three sequences, two from Texas (collected in May and October) and one from Brazil. This clade is heterogeneous, with high intraspecific diversity of 6.8% (figure 8). The genetic distance between the two clades is 12.2%.

The *Obelia dichotoma* dataset included 72 sequences, of which 40 were from the GoM, and 651 nucleotides, of which 295 (45.31%) were phylogenetically informative. The phylogenetic analyses show three well-supported clades (figure 9). Clade 1 contains sequences from northwest Atlantic (Rhode Island and Long Island, USA), southwest Atlantic (Uruguay) and three remaining sequences from the GoM. Clade 2 includes sequences from northwest Atlantic (Nova Scotia, Canada, Rhode Island and Massachusetts (USA)), southwest Atlantic (Argentina and Uruguay) and the Mediterranean Sea (Slovenia and Italy). Clade 3 contains most sequences from the GoM and three sequences from Brazil (Salinopolis). Regarding seasonality within the GoM, the three individuals of *O. dichotoma* found in clade 1 were collected in November and March, while the individuals in clade 3 were collected from September through November, with a minority from March and July. The genetic distance between the three clades ranged between 7.07% and 8.32% (figure 9).

Results from the species delimitation analyses are shown in the electronic supplementary material, tables S2–S4. The ABGD analyses indicate the presence of multiple clades within each nominal species (electronic supplementary material, table S2). In most partitions, the clades are congruent with the phylogenetic grouping shown in figures 1–9. Similarly, the best and second-best ASAP score identifies multiple cryptic species within each nominal species (electronic supplementary material, table S3). The results of species delimitation analyses performed in Geneious are reported in the electronic supplementary material, table S4. Most of the recognized species were well supported in at least one of the statistical tests (Rosenberg's P_AB_ and P(Randomly Distinct), P ID(Liberal) and P ID(strict)). Specifically, P(Randomly Distinct) is significant for most of the clades identified in figures 1–9 but not for all. Rosenberg's P_AB_ is significant for all clades, P ID(Liberal) is low for clade 1 in *Blackfordia virginica* only.

## 4. Discussion

Our data indicate that each of the eight species analysed in this paper show levels of crypsis across space, while six of the eight species analysed show level of sympatric crypsis (in Galveston, TX, GoM) and across seasons (table 1 for a summary). The genetic divergence between intraspecific lineages ranges between 3.6 and 12.99%, values generally considered consistent with the presence of cryptic species in Hydrozoa [17–20]. The majority of species delimitation analyses support the presence of cryptic species within the eight nominal species (see electronic supplementary material, tables S2–S4 and table S5 for a summary), with grouping similar to those shown in figures 1–9. Some analyses indicate a higher number of cryptic species than those reported here, thus suggesting that the numbers of cryptic species identified in figures 1–9 may be an underestimation.

**Table 1.** Nominal species investigated, number of cyptic lineages found worldwide, and number of lineages found within the GoM.

species investigated	cryptic lineages worldwide	number of lineages within the GoM
*Liriope tetraphylla*	4	1
*Lovenella assimilis*	5	4
*Nemopsis bachei*	3	2
*Eucheilota maculata*	3	2
*Ectopleura dumortierii*	5	2
*Malagazzia carolinae*	3	1
*Blackfordia virginica*	2	2
*Obelia dichotoma*	3	2
total	28	16

The two species that did not show cryptic lineages within the GoM are *Liriope tetraphylla* and *Malagazzia carolinae*. Historically considered a cosmopolitan species, the presence of cryptic species within *L. tetraphylla* had been hypothesized on account of the significant genetic difference between the mitochondrial 16S sequences from California and the Caribbean [26]. We confirm the presence of at least four cryptic lineages from the GoM and Caribbean, Pacific, North and South Atlantic, and Caribbean Sea, and a rather heterogeneous clade with specimens from China, South Africa and western Atlantic. More sequences are needed from most of the represented localities to assess the presence of additional cryptic species. Originally described from Florida, *Malagazzia carolinae* is the only *Malagazzia* species with range in the Atlantic Ocean and was only recently reported in the GoM [14]. We show that all the GoM sequences form one of three cryptic lineages, the other two being from China. Sequences from other regions where *M. carolinae* has been reported are not available.

All the other species analysed show sympatric cryptic lineages. *Nemopsis bachei* is considered native to the western Atlantic and the GoM but believed to be introduced in Europe [27]. It is also found in South America and China [20,28]. We show that in the GoM, there are two very distinct cryptic clades. While one clade appears during several months of the year, the other is more restricted to February and March, and appears rare in September (table 2). We also identify a third, distinct clade from China. Sequences from Europe are unavailable, and it is thus impossible to determine whether the accounts that *N. bachei* is introduced in German estuaries and beyond are supported. Because each clade in figure 2 harbours sequences from one location only, our dataset shows no indication that *N. bachei* is an introduced species in any of the localities from which sequences are available. *Lovenella assimilis* comprises five clades, four of which are in the GoM, all appearing at a different time of the year (although clades 3 and 5 briefly overlap in September) (table 2). Because very few sequences are available from the GoM and worldwide, more sampling is needed to clarify the status of the five clades represented in this study. *Eucheilota maculata* in the GoM is composed of two cryptic lineages appearing at different times (one in September and one in March and April). Despite the sampling efforts in the GoM, sequences available for *E. maculata* are scarce (both in the GoM and worldwide). Thus, similarly to *L. assimilis*, more sequences are needed to clarify the status of the three clades represented in figure 4. *Eucheilota* and *Lovenella* have a long and complicated taxonomic history, being historically put in different families based on morphology [29,30], but with more recent accounts showing minimal genetic distance between the two genera [31], and some evidence that species assigned to the two genera belong to the same species [25]. When combined, the two species dataset shows little resolution in the relationship between clades but clear indication that the two species are not reciprocally monophyletic (figure 5). *E. maculata* and *L. assimilis*, and more generally the two genera they belong to, need further taxonomic studies to determine whether they are separate taxonomic entities. *Ectopleura dumortierii* has a broad distribution, having been reported from the Mediterranean Sea, northeast Atlantic, northwest Atlantic and the GoM. We show that *E. dumortierii* in the GoM is in the form of two cryptic lineages, with different seasonality but some overlap in February (table 2). Additional cryptic lineages are from France and the United Kingdom (northeast Atlantic), an unknown location, and southeast Pacific (Chile). The individual from Chile shows a high divergence from the rest (about 12%), and further data are needed to validate this identification. *Blackfordia virginica* has long been considered a species introduced worldwide, with possible area of origin in the Black Sea. The species has been reported in western United States, India, China, Mexico, South America, Europe and Africa [27,32,33]. Our data identify a clade with specimens from several locations, including GoM (Texas and Louisiana), eastern and western United States (Virginia, Delaware, California), the Baltic Sea and China. This is consistent with the historical notion of B. *virginica* being introduced. However, a second small clade contains only three sequences identified as *B. virginica,* two from the GoM and one from Brazil. This clade shows rather conspicuous intraclade divergence. Our results partially challenge the notion that a single *B. virginica* is invasive worldwide in favour of a hypothesis of at least two cryptic lineages, one of which is potentially introduced in several locations. More sequences from regions such as Brazil, the Mediterranean Sea and Africa are needed to fully assess the claim that *B. virginica* is a globally introduced species. Our data also show that both lineages of B*. virginica* are present in the GoM (figure 8). Individuals from clade 1 were collected in May and October, while individuals from clade 2 were collected from February to July, with the highest concentration in May and June. This indicates that the two cryptic lineages were found together in May. Lastly, *Obelia dichotoma* is composed of at least three lineages. In the GoM, there are two cryptic lineages, one found in September to November, March and July, and the second found only (and overlapping with the first) in March and November.

**Table 2.** Monthly presence of cryptic lineages within each of the six nominal species that showed sympatric crypsis in the GoM. Numbers represent cryptic lineages as identified in figures 1–9. Months when two cryptic lineages temporally overlap are italicized.

Month	*Nemopsis bachei*	*Lovenella assimilis*	*Eucheilota maculata*	*Ectopleura dumortierii*	*Blackfordia virginica*	*Obelia dichotoma*
January						
February	*1; 3*			*2; 5*	2	
March	*1; 3*		3	2	2	*1; 3*
April	1	1	3		2	
May	1	4			*1; 2*	
June		4			2	
July		3				3
August		3				
September	*1; 3*	*3; 5*	1	2		3
October	1				1	3
November	1					*1; 3*

In summary, on a global scale, out of eight nominal species analysed, a total of 28 cryptic lineages were uncovered by our barcoding approach (table 1). These eight nominal species account for 28 cryptic lineages globally, indicating that the diversity is underestimated by a factor of 3.5. On a local scale, six species showed levels of crypsis within the sampled location. These six species accounted for a total of 16 lineages, indicating that the regional diversity is underestimated by a factor of 2. Cryptic species found in sympatry rarely overlap in time of appearance in the plankton and have different abundance peaks. The level of sympatric crypsis uncovered in this study is noteworthy because it implies that sampling in a single month of the year will rarely supply representatives of all the cryptic lineages present in a given location. This also implies that assessing a realistic diversity of an area needs a targeted sampling approach, which is costly and hard to achieve. Finally, our data reveal some cases in which cryptic species temporally overlap in the same location. This is also interesting as it raises questions on the evolution, speciation, and possible niche divergence within these co-occurring cryptic lineages.

The approach used in this study highlights a broader need to consider a too often overlooked time component that, when ignored, severely affects our capacity to find cryptic marine species in sympatry. It thus emphasizes the need for frequent, long-term sampling to evaluate local and global diversity. Our results, if generalized across taxa, might challenge the notion that sampling from a single location at a single time can capture representatives of a given nominal species. They also provide novel avenues to study the patterns and processes that led to the assemblages of sympatric cryptic species such as the ones highlighted in this study.

## 5. Conclusion

Using molecular barcoding, we show that eight nominal species of Hydrozoa are each composed of between 2 and 5 cryptic species each, for a total of 28 lineages. Of these 28 lineages, 16 were discovered in sympatry (Galveston, TX, Gulf of Mexico), where dense temporal sampling was conducted over a year. We show that cryptic species in the GoM appear in the same place (Galveston) at a different time of the year, adding a novel temporal component to the discovery of cryptic species.

Our data indicate that collecting only a few specimens as species representatives will underestimate the region's biodiversity, in our case study, by a factor of 2. Globally we find that the number of nominal species is 3.5 times smaller than the actual diversity identified by molecular barcoding. Overall, our data show that dense geographical and temporal sampling is necessary to estimate the diversity of a region and should be favoured over the most common procedure of sampling species at one time point from each locality of interest. Investing in temporally dense fieldwork and taxonomic training is paramount to the goal of assessing realistic biodiversity estimates.

## Data Availability

This paper has used already published sequences. GenBank accession numbers are reported in the electronic supplementary material, table S1, and below. GenBank accession nos. AY512510.1, U19377.1, MN355686.1, MN355682.1, MN355626.1, MN355691.1, MN355671.1, MN355674.1, MN355643.1, MN355613.1, MN355678.1, MN355681.1, MN355684.1,, MN355688.1, MN355635.1, MN355608.1, MN355675.1, MN355629.1, MN355690.1, MN355639.1, MH361353.1, MN355689.1, MN355687.1, MN355600.1, MN355634.1, MN355673.1, MN355610.1, MN355624.1, MN355648.1, MN355630.1, MN355621.1,MN355627.1, MN355663.1, MN355669.1, MN355685.1, MN355592.1, MN355575.1, MN355612.1, MN355590.1, MN355620.1, MN355597.1, MN355666.1, MN355645.1, MN355656.1, MN355618.1, MN355649.1, MN355602.1, MN355670.1, MN355628.1, MN355615.1, MN355662.1, MN355582.1, MN355640.1, MN355633.1, MN355587.1, MN355658.1, MN355577.1, MN355583.1, MN355654.1, MN355683.1, MN355653.1, MN355672.1, MN355664.1, MN355619.1, MN355655.1, MN355642.1, MN355625.1, MN355631.1, MN355623.1, MN355647.1, MN355637.1, MN355617.1, MN355638.1, MN355652.1, MN355660.1, MN355676.1, MN355614.1, MN355616.1, MN355657.1, MN355596.1, MN355667.1, MN355668.1, MN355680.1, MN355632.1, MN355584.1, MN355677.1, MN355594.1, MN355641.1, MN355576.1, MN355591.1, MN355601.1, MN355622.1, MN355595.1, MN355659.1, MN355665.1, MN355661.1, MN355679.1, MN355606.1, MN355636.1, MN355650.1, MN355609.1, MN355605.1, MN355611.1, MN355604.1, MN355599.1, MN355578.1, MN355579.1 MN355580.1, MN355581.1, MN355585.1, MN355589.1, MN355598.1, MN355607.1, MN355644.1. MN355646.1, MN355586.1, MN355593.1, MN355651.1, MN355603.1, MN355588.1, EU999222.1, EU293980.1, KT266624.1, KY077290.1, MG979365.1, JQ715945.1, KJ859220.1, JQ715944.1, MN341844_1, MN341852_1, MN341854_1, MN341847_1, MN341848_1, MN341849_1, MN341850_1, MN341851_1, MN341853_1, MN341845_1. MN341846, KR263041_1, KR263037_1, KR263038_1, KR263039_1, KR263040_1, MN355881.1, MN355885.1, MN355862.1, MN355889.1, MN355890.1, MN355888.1, MN355847.1, MN355880.1, MN355882.1, MN355886.1, MN355883.1, MN355894.1, MN355887.1, MN355892.1, MN355904.1, MN355902.1, MN355884.1, MN355850.1, MN355903.1,. MN355877.1, MN355897.1, MN355896.1, MN355863.1, MN355849.1, MN355891.1 MN355893.1, MN355895.1, MN355901.1, JQ715889.1, JQ715898.1, JQ715901.1, JQ715895.1, JQ715896.1, JQ715897.1, JQ715899.1, JQ715900.1, MN355873.1, MN355875.1, MN355866.1, MN355858.1, MN355872.1, MN355876.1, MN355861.1, MN355865.1, MN355869.1, MN355852.1, MN355848.1, MN355867.1, MN355851.1, MN355860.1, MN355855.1, MN355854.1, MN355868.1, MN355879.1, MN355857.1,MN355846.1, MN355856.1, MN355874.1, MN355878.1, MN355870.1, MN355871.1, MN355859.1, MN355864.1, MN355853.1, MN355899.1, MN343754.1, MN343755.1, MN343756.1, MN367055.1, MN343757.1, MN343758.1, KT266613.1, FJ550501.1, KY363942.1, KX355420.1, EU876534.1 FN687542.1, FN687543.1, MN355704.1, MN355697.1, MN355694.1. MN355707.1, MN355695.1, MN355710.1, MN355708.1, MN355696.1, MN355712.1, MN355711.1, MN355717.1, MN355716.1, MN355715.1, MN355713.1, MN355714.1, EU305474.1, MN355698.1, MN355703.1, MN355692.1, MN355693.1, MN355709.1, MN355706.1, MN355705.1, MN355699.1, MN355700.1, MN355701.1, MN355702.1, JQ715904.1, JQ715905.1, KF962491.1, KF962492.1, KF962493.1, KF962494.1, KF962495.1, KF962496.1, KF962498.1, KF962497.1, KF962499, MN355770.1, MN355771.1, MN355772.1, MN355773.1, MN355774.1, MN355775.1, MN355776.1, MN355777.1, MN355778.1, MN355779.1, MN355780.1, MN355781.1, MN355782.1, MN355783.1, MN355784.1, MN355785.1, MN355786.1, MN355787.1, MN355788.1, MN355789.1, MN355790.1, MN355791.1, MN355792.1, MN355793.1, MN355794.1, MN355795.1, MN355796.1, MN355797.1, MN355798.1, MN355799.1, MN355800.1, MN355795.1, MN355802.1, MN355803.1m MN355804.1, MN355805.1, MN355806.1, MN355807.1, MN355808.1, MN355809.1, MN355810.1, MN355811.1, MN355812.1, MN355807.1, MN355814.1, MN355815.1, MN355816.1, MN355817.1, MN355818.1, MN355819.1, MN355820.1, MN355821.1,MN355822.1, MN355823.1, MN355824.1, MN355825.1, MN355826.1, MN355827.1, MN355828.1, MN355829.1, MN355814.1, MN355831.1, MN355832.1, MN355833.1, MN355834.1, MN355835.1, MN355836.1, MN355837.1, MN355838.1, MN355832.1, MN355840.1, MN355841.1, MN355842.1, MN355843.1, MN355844.1, MN355845.1, AY512516, MH460957, KC507381, KC507380, KC507379, KC507378, KC507374, KC507373, KC507372, KC507370, KC507369, KC507368, MN355949, MN355948, MN355947, MN355946, MN355944, MN355945, MN355943, MN355942, MN355941, MN355940, MN355939, MN355938, MN355937, MN355936, MN355935, MN355933, MN355934, MN355932, MN355931, MN355930, MN355929, MN355928, MN355926, MN355927, MN355925, MN355924, MN355923, MN355922, MN355921, MN355920, MN355919, MN355918, MN355917, MN355916, MN355915, MN355914, MN355913, MN355912, KT266605, KF962405, KF962404, KF962403, KF962402, KF962401, JQ715957, JQ715956, JQ715955, JQ715954, JQ715953, JQ715952, MT849350, MN355768, MN355767, MN355766, MN355765, MN355764, MN355763, MN355762, MN355761, MN355760, MN355759, MN355758, MN355757, MN355756, MN355755, MN355754, MN355753, MN355752, MN355751, MN355750, MN355749, MN355748, MN355747, MN355746, MN355745, MN355744, MN355743, MN355742, MN355741, MN355740, MN355739, MN355738, MN355737, MN355736, MN355735, MN355734, MN355733, MN355732, MN355731, MN355730, MN355729, MN355728, MN355727, MN355726, MN355725, MN355724, MN355723, MN355722, MN355721, MN355720, MN355719, MN355718, MG786561, KX665360, KX665359, KX665347, KX665345, KX665342, KX665331, KX665330, KX665326, KX665325, KX665324, KX665322, KX665316, KX665309, KX665297, KX665288, KM603472. Seasonality data are reported in [14] and publically available here: https://peerj.com/articles/7848/. Collecting data are also reported in the same paper: https://peerj.com/articles/7848/. Raw data from [14] can be found here: 10.7717/peerj.7848/supp-1. Additional information is provided in the electronic supplementary material [34].
